# Electroacupuncture at ST36 Alleviates Visceral Hypersensitivity by Suppressing Eosinophil‐Induced TRPV1 Expression on Duodenum

**DOI:** 10.1155/prm/5547436

**Published:** 2026-04-03

**Authors:** Yue-Jie Li, Lu-Ping Liu, Na-Na Yang, Jing-Wen Yang, Cun-Zhi Liu

**Affiliations:** ^1^ International Acupuncture and Moxibustion Innovation Institute, School of Acupuncture-Moxibustion and Tuina, Beijing University of Chinese Medicine, Beijing, 100029, China, bucm.edu.cn; ^2^ School of Acupuncture-Moxibustion and Tuina, Shandong University of Traditional Chinese Medicine, Jinan, Shandong, China, sdutcm.edu.cn

**Keywords:** electroacupuncture, enteric neuron, eosinophil, TRPV1, visceral hypersensitivity

## Abstract

**Background and Aims:**

Electroacupuncture (EA) has been reported to have a therapeutic potential for visceral hypersensitivity. Recent studies have highlighted the crucial role of the duodenum in visceral hypersensitivity, suggesting it as a potential therapeutic target. This study investigates how EA regulates visceral hypersensitivity in the duodenum.

**Method:**

We established a visceral hypersensitivity model in rats by intragastric administration of iodoacetamide. Various parameters were evaluated, including electromyographic recordings in vivo, mesentery afferent nerve spontaneous discharge, mechanical sensitivity, and chemical sensitivity. Western blotting and immunofluorescence were employed to detect the protein expression of nociceptor transient receptor potential vanilloid 1 (TRPV1) and to assess eosinophil infiltration by labeling eosinophil major basic protein (EMBP). We examined whether the effect of EA was mediated by TRPV1, using the agonist capsaicin and the antagonist SB366791. Eosinophils were counted, and their activation and degranulation status were assessed. We explored the impact of eosinophils on the neuron fibers of the intestine.

**Results:**

EA decreased firing of visceral afferent action potentials including spontaneous, mechanosensitive and chemosensitive mesentery afferent discharge, and attenuated visceromotor electromyographic activity. The above effects of EA were reversed by intraperitoneal injection or in vitro enteric nerve administration of TRPV1 agonist capsaicin. EA substantially reduced the area of neuron fibers and the TRPV1 expression. Furthermore, in vitro experiments using primary cell cultures indicated that eosinophils’ degranulation with different doses could stimulate neuron fibers. EA at ST36 was found to reduce both the number of duodenal eosinophil‐positive cells and the levels of their degranulation products.

**Conclusion:**

These results suggested that EA at ST36 alleviates visceral hypersensitivity by inhibiting eosinophils degranulation and TRPV1 expression in the duodenum.

## 1. Introduction

Functional dyspepsia (FD) is a common upper gastrointestinal disorder causing symptoms like abdominal pain, bloating, and early satiety, without any identifiable organic cause [1]. One of the key aspects of FD is visceral hypersensitivity, a phenomenon where normal physiological stimuli are perceived as painful due to an abnormal increase in nerve sensitivity [2–5]. Several studies have shown that while targeted therapies do not work for everyone and often come with notable side effects that can compromise patient adherence, they may provide relief for some patients with visceral hypersensitivity.

The transient receptor potential vanilloid 1 (TRPV1) receptor plays a significant role in regulating visceral sensitivity and is pivotal in the pain perception process [6]. Abnormalities in the duodenum, such as low‐grade inflammation, increased mucosal permeability, and hypersensitivity of duodenal mucosa, may induce dyspeptic symptoms [2–5]. It is postulated that duodenal inflammation may directly stimulate submucosal nerve endings by activating nociceptors [7]. Emerging evidence implicates eosinophils as potential key players in the pathophysiology of functional gastrointestinal disorders. Studies have reported a significant increase in eosinophil counts in the duodenal mucosa of FD patients compared to healthy controls, and this infiltration correlates with symptom severity, especially early satiety and postprandial distress. Beyond mere presence, activated eosinophils release cytotoxic granule proteins—such as major basic protein (MBP) and eosinophil cationic protein (ECP)—as well as inflammatory mediators (e.g., cytokines and nerve growth factor). These substances can directly impair mucosal barrier integrity, activate sensory nerve endings (including those expressing TRPV1), and promote visceral hypersensitivity, a central feature of FD [8, 9]. The intricate interplay within the neuroimmune system in the duodenum of patients with FD has prompted speculation regarding the potential involvement of inflammatory cells, particularly eosinophils and their mediators, in the pathophysiology of this condition [10, 11].

EA has emerged as a promising therapeutic intervention for managing functional gastrointestinal disorders [12]. Several clinical studies have elucidated the efficacy of acupuncture in alleviating gastrointestinal symptoms, such as abdominal pain, early satiety, and post‐prandial fullness [13, 14]. EA has been shown to promote various physiological responses, including increased release of endogenous opioids, modulation of inflammatory mediators, and restoration of gut motility [15, 16]. Recent studies highlight the efficacy of EA in reducing visceral hypersensitivity, suggesting a multifaceted mechanism of action [17]. EA appears to reduce visceral hypersensitivity in FD through multiple mechanisms, including modulation of neurotransmitter release, interaction with TRPV1 receptors, reduction of inflammatory mediators, activation of central nervous system pathways, restoration of gut–brain axis function, and alleviation of stress‐related symptoms [18]. However, the prior research on acupuncture for the treatment of visceral hypersensitivity has notable limitations, particularly in its failure to adequately address the underlying causes of inflammation and pain. Existing studies primarily focus on the symptomatic relief provided by acupuncture, often overlooking the critical role that inflammatory processes play in the development of visceral hypersensitivity. In this study, we aimed to investigate the effects of EA on visceral hypersensitivity and the associated mechanisms in a rat model of FD. We hypothesized that EA would alleviate visceral hypersensitivity by reducing the expression of TRPV1 receptors induced by eosinophil degranulation in the duodenum.

## 2. Materials and Methods

### 2.1. Animals

Sprague‐Dawley rat dams with their litters of pups (10–12 male pups/dam) were acquired from Beijing Vital River Laboratory Animal Technology Co., Ltd. All animals received ad libitum access to standard laboratory chow and water. Animals were acclimatized for no less than 72 h post arrival prior to inclusion. Housing parameters included controlled ambient temperature (22°C–24°C), relative 50%–60% humidity, and a 12‐h light/dark cycle. At 3 weeks, male animals were housed four per cage. All animal experiments were approved by the Institutional Animal Care and Use Committee (BUCM‐4‐2022021803‐1021).

Euthanasia for fresh tissue collection: rats were anesthetized by inhaling a mixture of 5% isoflurane and oxygen until the foot reflexes were lost, confirming deep anesthesia. Then, while the animals remained in a deep anesthesia state, a sterile rodent decapitation device was used for decapitation. This method ensured that the tissues could be immediately fixed for molecular analysis.

Euthanasia perfusion protocol: the rats were given terminal anesthesia, and 5% isoflurane was administered until all brainstem reflexes (corneal reflex and pain response reflex) disappeared. Under a state of continuous deep anesthesia, perfusion was initiated through the left ventricle. 37°C normal saline was used first, followed by the addition of 4% paraformaldehyde. Cardiac arrest caused by the perfusion occurred within 30 s, and no vital signs could be confirmed as death occurred more than 5 min after the operation. All animal experiments were approved by the Institutional Animal Care and Use Committee (BUCM‐4‐2022021803‐1021).

### 2.2. FD Model

Studies [19] have shown that the FD model could be prepared by intragastric administration of iodoacetamide (IA). The 10‐day‐old SD male rats were randomly allocated into a control group and a model group. The rats in the control group were orally administered a 2% sucrose solution at a dose of 0.2 mL/d for six consecutive days, and the rats in the model group were given gavage a mixture of 0.1% IA and 2% sucrose 0.2 mL/d. Model group animals were randomized into FD, EA, and SEA experimental groups.

### 2.3. EA Treatment

Animals were anesthetized by 1.5% inhaled isoflurane via an anesthesia machine and thermally supported using a heating pad to prevent hypothermia. Each group of animals received the same amount of anesthesia. Daily EA stimulation was delivered continuously for 30 min at parameters of 1‐mA current intensity, 100‐Hz frequency, and a pulse width 0.1 ms using the HANs‐200A Acupuncture Point Nerve Stimulator (Nanjing, China), for 10 days in a row. Stainless steel acupuncture needles (0.16∗13 mm) were inserted bilaterally at ST36 (Zusanli) at 5‐mm depth. For SEA, stainless steel needles were shallowly inserted at bilateral ST36 without connecting to the HANs‐200A. The ST36 acupoint is anatomically situated approximately 5 mm distal to the knee joint, 2 mm lateral to the anterior tubercle of the tibia, adjacent to the common peroneal and tibial nerve branches of the sciatic nerve [20].

### 2.4. Mesentery Afferent Nerve Activity In Vitro

On the second day of EA treatment, rats were anesthetized by 3% inhaled isoflurane. A proximal intestinal segment with attached mesentery was rapidly excised after a midline laparotomy. The segment was immersed in 4°C precooled Krebs solution, and the intestinal contents were irrigated. The gastrointestinal segment was secured in a custom perfusion chamber and continuously perfused with oxygenated Krebs solution (10 mL/h, 37°C) maintained at a constant temperature. The segment was positioned in an organ bath with its oral end cannulated to a syringe pump for controlled luminal perfusion and its aboral end linked to an outflow conduit. Mesenteric fixation was achieved using insect pins securing the tissue to the chamber. Using ophthalmic forceps under stereomicroscope visualization, we gently dissected adipose tissue from the mesentery with traction, avoiding injury to embedded vasculature and afferent neural structures. The isolated and cleaned intestinal nerve was separated from the adipose tissue layer, and the efferent end was cleanly transected using surgical scissors to ensure a smooth and even cut. Position the suction electrode adjacent to the severed afferent nerve stump, then draw the entire nerve into the glass capillary. The signal was viewed as spikes on the computer using LabChart8.0 software. The baseline refers to the spontaneous nerve discharge observed in a resting state prior to any mechanical or chemical stimulation. Ramp distention refers to the mechanosensitivity of mesentery afferent nerve discharge with gradient pressure expansion. The effect of capsaicin on spontaneous discharge of the mesenteric afferent nerves was observed by adding capsaicin (1 μg/section, APEbio) to the organ bath. The capsaicin solution was added to the organ bath, and the mesenteric nerve was immersed in the test solution containing the capsaicin solution.

### 2.5. Electromyographic Recording

Rats underwent 18 h fasting prior to anesthesia induction with 1.5% isoflurane. A latex condom (2.5 cm) was connected to a polyethylene tube (PE240) and inserted into the rats’ stomach through the mouth. Platinum wire electrodes were implanted into the left acromiotrapezius muscle. The condom catheter was connected to a barostat, while the electrodes were attached to a biological system for electromyograms (EMG) recording. The rat was subjected to varying intragastric pressures (20, 40, 60, 80 mmHg) by inflating a balloon for 30 s, with a 3‐min gap between each distension. The EMG was recorded by the LabChart8.

### 2.6. Drug Administration

Rats were intraperitoneally injected with the TRPV1 agonist capsaicin (0.3 mg/kg, APEbio) or the selective TRPV1 antagonist SB‐366791 (0.3 mg/kg, APEbio). The drugs were suspended in 1% Tween 80 and 1%DMSO in phosphate‐buffered saline. The EMG was observed individually after the i.p. administration of capsaicin or SB366791 for 30 min.

### 2.7. Western Blot

The duodenal tissue from anesthetized rats was quickly removed and washed in PBS. The protein samples were extracted and prepared on ice with RIPA buffer containing protease inhibitors. The liquid supernatant was collected and estimated for protein concentration using Thermo solution kit after centrifuging at 14,000 r/min for 30 min at 4°C. Equal protein quantities were resolved by SDS‐PAGE and electrophoretically transferred (300 V, 1 h) to 0.45 μm PVDF membranes. Membranes were blocked with 5% nonfat milk in TBST (Tris‐buffered saline containing 0.05% Tween‐20). Target proteins were identified by specific primary antibodies against EMBP (sc‐365701, SantaCruz; 1:500), TRPV1 (GTX54762, GeneTex; 1:500). Anti‐mouse HRP (1:5000) and anti‐rabbit HRP (1:5000) were used for secondary antibodies from Cell Signaling Technology. The bands were visualized by enhanced chemiluminescence and quantified using ImageJ.

### 2.8. Fluorescent Immunostaining

Anesthetized rats were transcardially perfused with 4% paraformaldehyde solution. The duodenum was soaked in 4% PFA overnight and then immersed in 20% and 30% sucrose solution for 2 days at 4°C. Cryosections (10 μm) were prepared from frozen intestinal blocks. Sections were permeabilized with 0.1% Triton X‐100 (15 min) and incubated in blocking buffer for 1 h at 37°C. After blocking, intestinal slices were incubated with primary antibody EMBP (sc‐365701, Santa Cruz; 1:500)\TRPV1 (GTX54762, GeneTex; 1:500)\HuC/D (ab210554, abcam; 1:1000)\PGP9.5 (ab8189, abcam; 1:1000) at 4°C overnight. Secondary antibodies incubation proceeded for 2 h at 37°C. For Carbol 2R staining, sections were incubated with Carbol 2R for 10 min and hematoxylin for 3 min. For cell fluorescent staining, cell samples were fixed with 4%PFA after culture and collection. Then, they underwent fluorescence immunostaining following the above steps. The fluorescence images were acquired by Olympus and analyzed in ImageJ and Angio Tool software. The average of 6 images was randomly captured and calculated per rat. Analysis of fluorescence images was performed by two unrelated individuals.

### 2.9. Cell Culture Experiment

Enteric neurons were isolated as described previously [21, 22]. Rats were anesthetized and euthanized, and laparotomy was performed. Based on a protocol published by Smith, the duodenum was removed and lavaged, and the longitudinal muscle with the adherent myenteric plexus was dissected. Tissue digestion was performed in digestion buffer (consisting of 5 mg collagenase IV Thermo Fisher) and 0.5 mg DNase I in 5 mL of DMEM/F‐12 medium (30 min) at 37°C and 300 rpm. Tissue underwent gentle trituration and 100 μm filtration. The cells were resuspended in 1 mL of planting medium (containing 900 μl Neurobasal‐A medium), 100 μL of fetal bovine serum, 10 μL of penicillin‐streptomycin, and 0.5 mM L‐glutamine in each of the 12 wells. The neuron culture was incubated under standard conditions at 37°C with 5% CO_2_ for 5.5 h (The enteric neurons began to stick to the wall after 4 h culture, while smooth muscle cells did not start to stick to the wall until 6 h later) to clear smooth muscle cells. The cells were resuspended in 1 mL of complete neuron media (containing 980 μl Neurobasal‐A medium, 20 μL B‐27 supplement, 10 μL penicillin‐streptomycin, and 2 mM L‐glutamine) in each of the 12 wells, and neurons were incubated, with the culture medium replaced every 48 h. Enteric neurons become viable for EMBP stimulation after 8 days in vitro.

EMBP stimulation. After 8 days culture, 10, 100, and 1000 ng/mL recombinant murine was added to EMBP in 1 mL of complete neuron media to stimulate enteric neurons, separately. Neuron was incubated at 37°C with 5% CO_2_ for 36 h. In the control group, ddH_2_O incomplete neuron media was added to culture neuron.

After stimulation, a precoated glass coverslip was taken off from 12‐well culture and then fixed with 4% PFA. After washing in PBS, the coverslip was permeabilized in 0.5% Triton‐100 (30 min) at RT and then blocked (30 min) in 5% bovine serum albumin at 37°C. Antibody‐tubulin (1:1000, abcam) was incubated for 1 day at 4°C and incubated with secondary antibody (1:1000, abcam) for 1 h at RT.

### 2.10. Statistical Analysis

All statistical analyses were performed using GraphPad 8. Data were presented as mean ± SEM. We tested data for normality using the Shapiro–Wilk test and for homogeneity of variances using Levene’s test. For comparisons across multiple groups, we used one‐way ANOVA with Tukey’s post hoc test for normally distributed data and the Kruskal–Wallis test with Dunn’s post hoc test for non‐normally distributed data. We defined statistical significance as a *p*‐value < 0.05.

## 3. Results

### 3.1. EA at ST36 Alleviated the Visceral Hypersensitivity in FD Rats

To evaluate the downregulatory effect of visceral hypersensitivity by EA on FD rats in vivo, EMG was measured for muscle contraction analyses (Figure 1(a)). A gradually and slightly increased signal of EMG under a pressure gradient was observed in the control group. The FD group displayed a rapid and notable response of EMG from 20‐mmHg pressure, which continued to amplify with increasing pressure, suggesting that a visceral hypersensitivity was induced in FD rats. EA treatment at ST36 significantly suppressed the visceral hypersensitivity in response to both physiological (20 mmHg) and noxious (40, 60, 80 mmHg) stimuli, while SEA reduced response of EMG within a limited pressure range (40 and 60 mmHg) (Figures 1(b) and 1(c)). These results indicated that to obtain a powerful effect on visceral hypersensitivity in FD rats, EA but not SEA treatment at ST36 are necessary.

FIGURE 1EA ST36 alleviated the visceral hypersensitive in FD rats. (a) Schematic depicting that model of the pressure probe and distention balloon used to record EMG. (b) Representative pressure (top, red) and EMG (bottom, green) recordings. (c) The area under the curve (AUC) was used to quantify the EMG. ^∗^ *p* < 0.05; ^∗∗^ *p* < 0.01; ^∗∗∗^ *p* < 0.001. *n* = 11 per group. (d) Schematic showing the mesentery afferent nerve experiment. (e) Representative images of spontaneous discharge from four groups. (f) The average 2 min of spontaneous discharge frequency. ^∗^ *p* < 0.05; ^∗∗^ *p* < 0.01; ^∗∗∗^ *p* < 0.001. *n* = 6 per group. (g) Representative images of ramp distension from four groups. (h) The average 2 min of distension discharge frequency at different pressures. ^∗^ *p* < 0.05; ^∗∗^ *p* < 0.01; ^∗∗∗^ *p* < 0.001. *n* = 6 per group. (i) The max of distension discharge frequency at different pressures.

**Figure:**
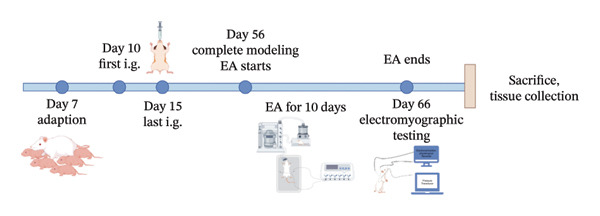
(a)

**Figure:**
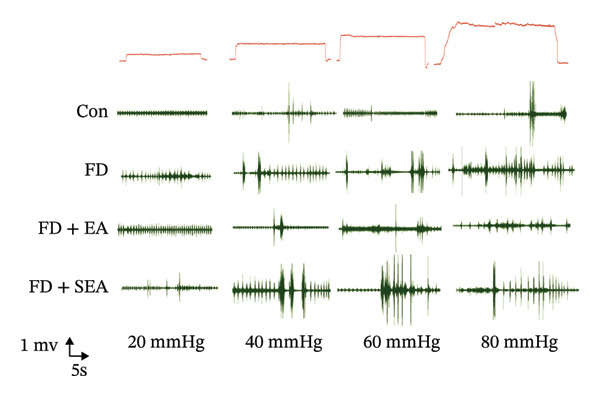
(b)

**Figure:**
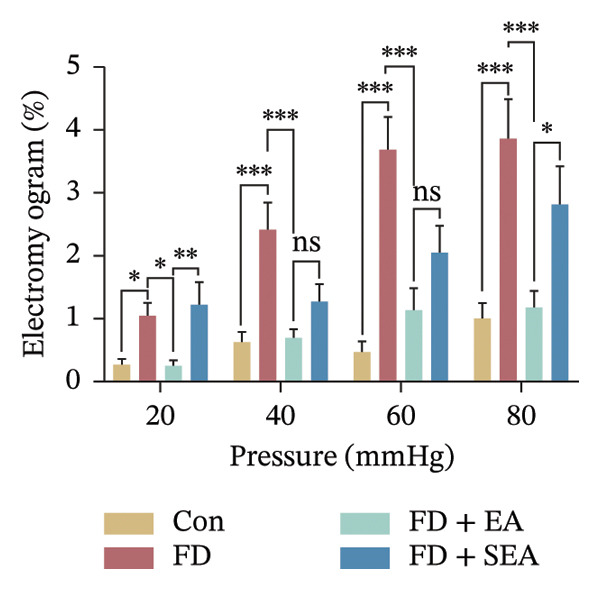
(c)

**Figure:**
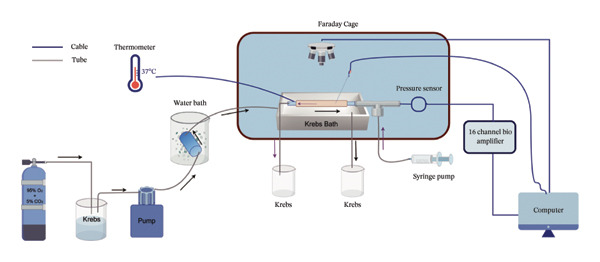
(d)

**Figure:**
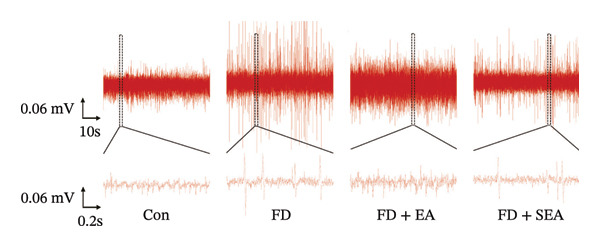
(e)

**Figure:**
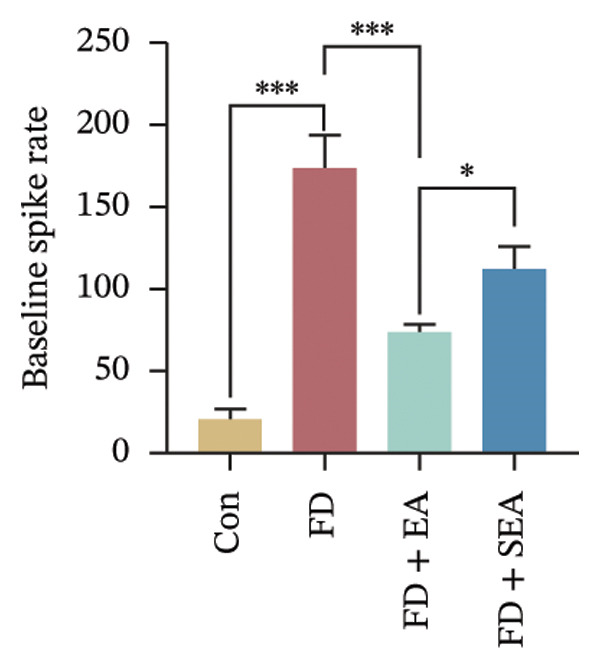
(f)

**Figure:**
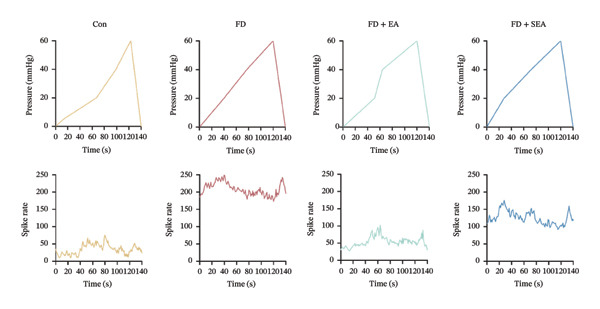
(g)

**Figure:**
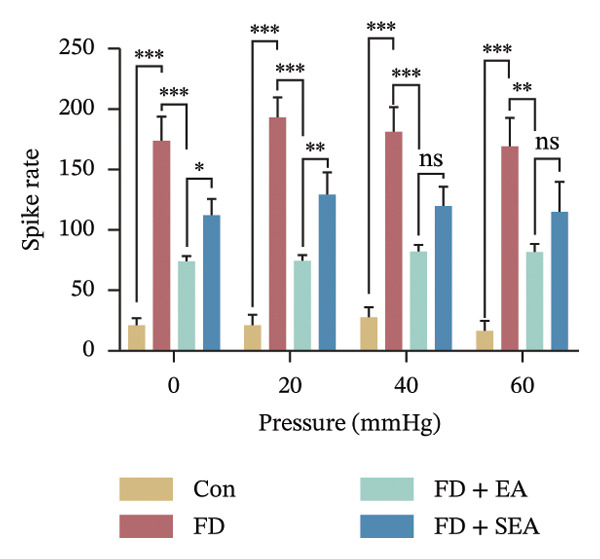
(h)

**Figure:**
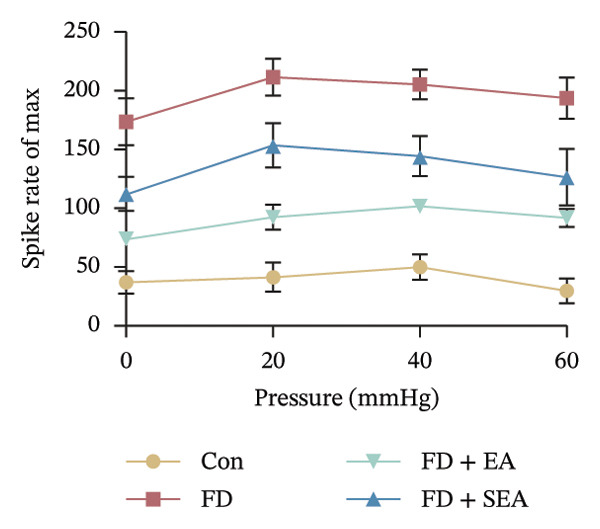
(i)

We observed the impact of EA on visceral hypersensitivity by recording mesentery afferent nerve discharge in vitro including the data of baseline and ramp distention (Figure 1(d)). Mesenteric afferent nerves exhibited consistent and uninterrupted spontaneous discharge (Figures 1(e) and 1(f)). In the FD group, the mean afferent firing rate was elevated fivefold compared to the Con group at baseline. Both EA and SEA decreased spontaneous discharge. Indeed, the spontaneous discharge of EA reduced profound 57% compared to the spike observed in the FD group, while the SEA’s spontaneous discharge exhibited a reduction (37%) (Figure 1(f)). A significant difference was observed between the EA group and the SEA group. Mesenteric afferent baseline activity showed significant attenuation following long‐term EA, indicating persistent suppression of spontaneous discharge (Figure 1(f)).

Data on intestinal ramp distension obtained from the four groups were presented, demonstrating a progressive increase in spike rate at different volumes of distension (Figure 1(g)). The relationship between pressure and spike rates revealed an increase in mesentery afferent nerve discharge during various levels of distention. The Con group produced slight mesentery afferent nerve discharge (the discharge frequency of all four groups was less than 50 imp/s), while the FD group displayed a notable increase in response to nerve discharge at 20, 40, and 60‐mmHg pressure (the discharge frequency of all four groups was more than 150 imp/s) (Figure 1(h)). EA treatment was sufficient to trigger significant decrease ramp distention stimulation, indicating a decreased pain sensitivity to visceral stimulation (Figure 1(h)). The SEA group also exhibited reduced mesentery afferent nerve discharge at pressures of 40 and 60 mmHg (Figure 2(e)).

FIGURE 2The inhabitation effects of EA on visceral hypersensitivity were achieved by suppressing TRPV1. (a and b) Quantification of local TRPV1 expression within the rat duodenum by western blotting. ^∗^ *p* < 0.05; ^∗∗^ *p* < 0.01; ^∗∗∗^ *p* < 0.001. *n* = 3 per group. (c) Representative images of immunolabeling for nociceptor (TRPV1, red) and enteric neuron (PGP9.5, green) in the enteric system of four groups. Scale bars, 20 μm, 40X. (d) Representative images of immunolabeling for enteric neuron (PGP9.5, red) and enteric neuron body (labeled with HuC/D, green) in the enteric system of the FD group. Scale bars, 50 μm, 20X. (e) Representative images showing immunolabeling for PGP9.5 in rat duodenum. Scale bars, 200  μm,4X. (f) The area of PGP9.5 in duodenum. ^∗^ *p* < 0.05; ^∗∗^ *p* < 0.01; ^∗∗∗^ *p* < 0.001. *n* = 3 per group. (g) Representative images of discharge following capsaicin from four groups. (h) The total of spike rate with capsaicin stimulation. ^∗^ *p* < 0.05; ^∗∗^ *p* < 0.01; ^∗∗∗^ *p* < 0.001. *n* = 3 per group. (i) Representative pressure (top, red) and EMG (bottom, green) recordings after injecting TRPV1 agonist (capsaicin) or antagonist (SB366791). (j) The area under the curve (AUC) was used to quantify the EMG. ^∗^ *p* < 0.05; ^∗∗^ *p* < 0.01; ^∗∗∗^ *p* < 0.001. *n* = 8 per group.

**Figure:**
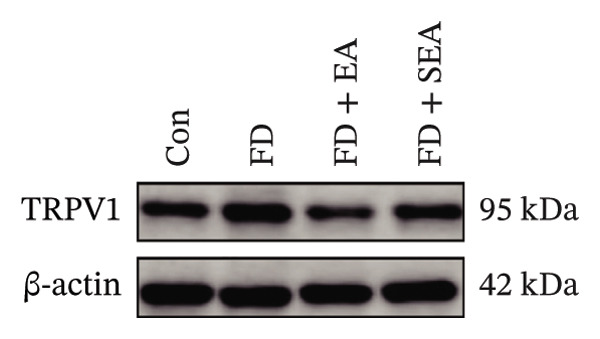
(a)

**Figure:**
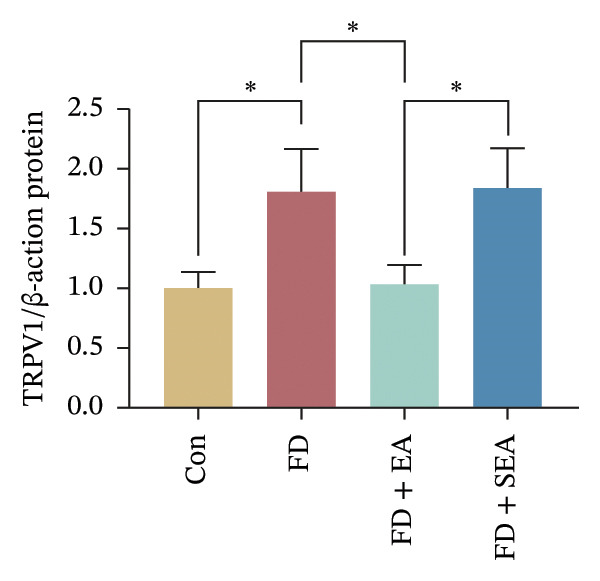
(b)

**Figure:**
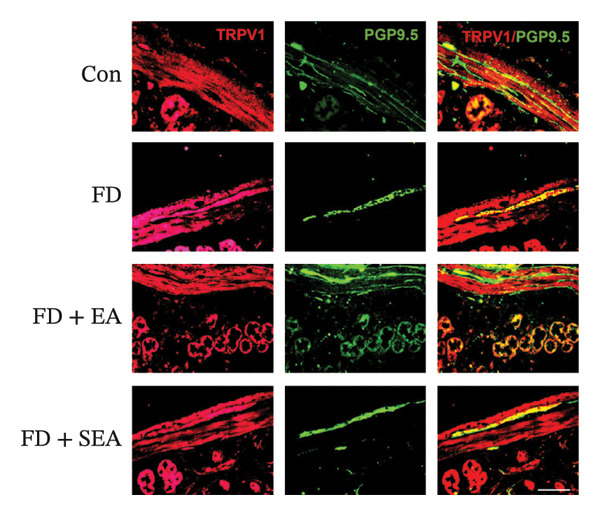
(c)

**Figure:**
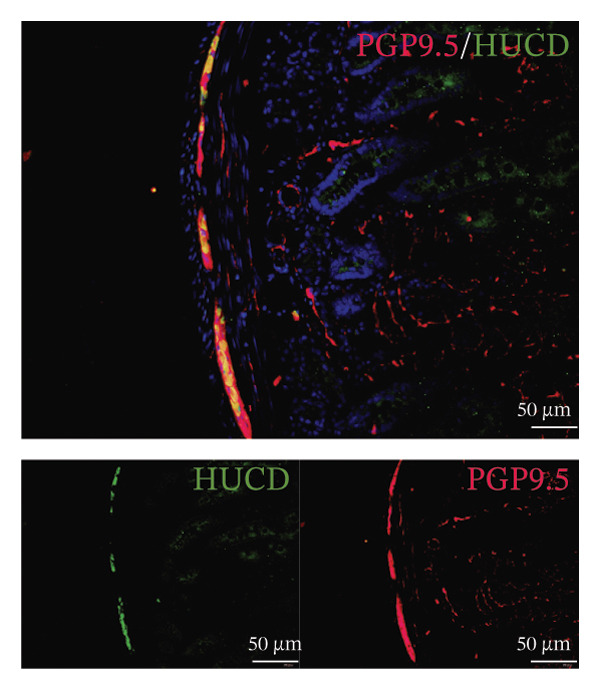
(d)

**Figure:**
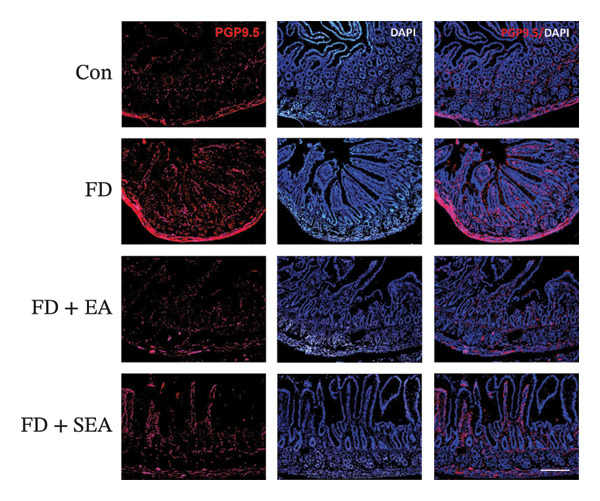
(e)

**Figure:**
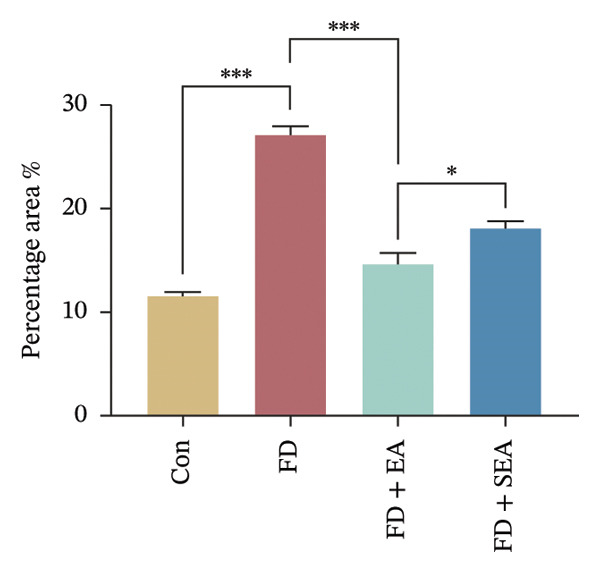
(f)

**Figure:**
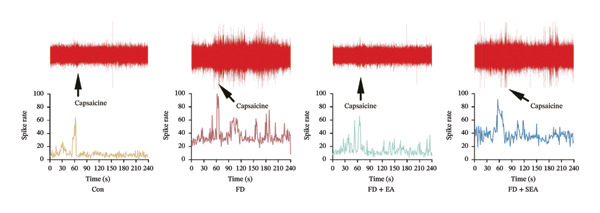
(g)

**Figure:**
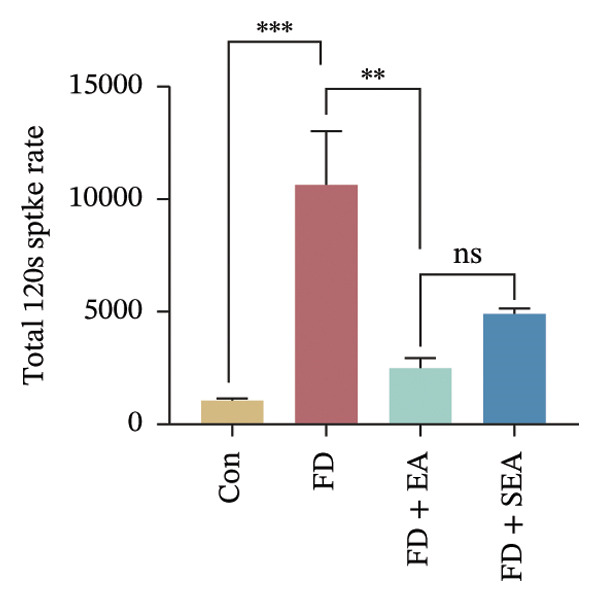
(h)

**Figure:**
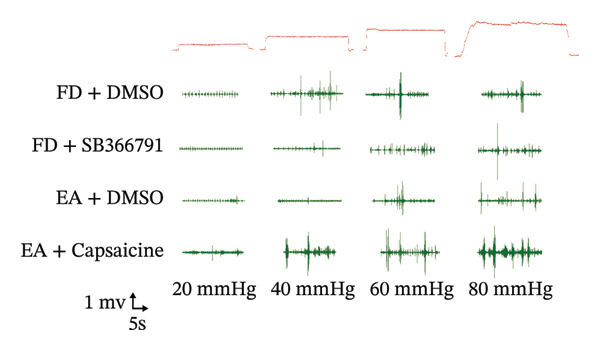
(i)

**Figure:**
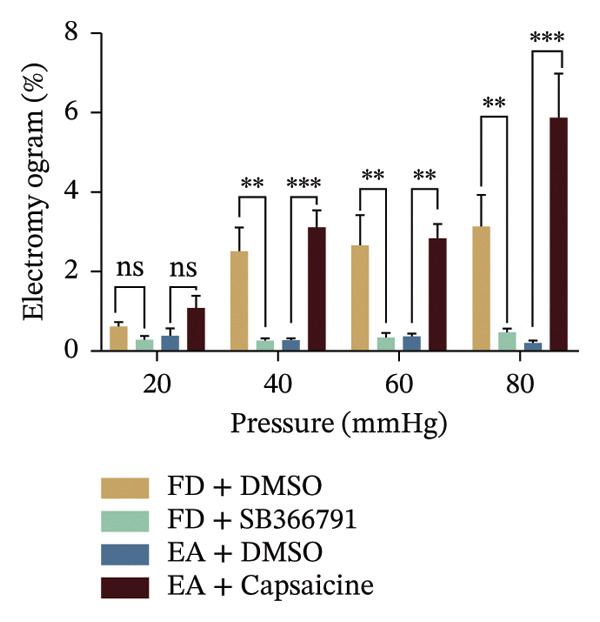
(j)

Notably, a disproportionately higher spike discharge level was observed at gradient distending pressures (Figure 1(i)). The maximum spike rates peaked at 20‐mmHg pressure in the FD group (220 imp/s) and the SEA group (150 imp/s), while the maximum spike rates peaked at 40‐mmHg in the Con group (50 imp/s) and the EA group (100 imp/s) (Figure 1(i)). The study found that the mesenteric afferent nerves in the EA group exhibited decreased discharge in response to ramp distention, indicating a reduction in mechanical sensitivity following EA treatment.

### 3.2. EA at ST36 Reduced the TRPV1 Expressed on Duodenum

Enteric neurons established both anatomical proximity and functional interactions with TRPV1‐expressing nociceptors. The IA challenge resulted in a significant upregulation of TRPV1 protein expression in the FD group, with data showing higher levels of TRPV1 compared to the Con group (Figures 2(a) and 2(b)). The elevated TRPV1 content was attenuated in the EA treatment, whereas the SEA did not reduce the TRPV1 content. We suppose that the increase in TRPV1 synthesis contributed to heightened sensitivity of neurons and subsequent excitation of visceral afferent nerves. TRPV1 undergoes neuronal synthesis followed by axonal trafficking to terminals through axoplasmic transport. While immune cells have been shown to express TRPVs, our immunofluorescence revealed selective TRPV1 overexpression within the myenteric nervous plexus (Figure 2(c)).

Whereas most enteric neuron body expression is confined to the myenteric nervous plexus, we used two different markers, PGP9.5 and HuC/D, to observe the distribution of neurons and their fibers throughout the duodenum, allowing for the evaluation of nerve terminal activity in the enteric nervous system (Figure 2(d)). Enteric neuron bodies marked by the expression of HuC/D are the same as those labeled by PGP9.5, whereas PGP9.5 also marked the nerve terminal in the whole intestine. We revealed an increase of 16% in the density of neuron area in the duodenum of the FD group. The density of neuron area in the EA group was reduced nearly 13% from that observed in the FD group (Figures 2(e) and 2(f)).

### 3.3. EA at ST36 Improves Visceral Hypersensitivity Through Suppressing TRPV1

It is noteworthy that TRPV1 is predominantly expressed by visceral afferents and is present in the majority of afferents innervating the gastrointestinal tract. Consequently, the study investigated the sensitivity of mesenteric afferent nerves to capsaicin (a TRPV1 agonist) by applying capsaicin to the serosal surface of the intestine (Figure 2(g)). Capsaicin elicited a rapid increase in mesenteric afferent discharge, peaking within 60 s before gradually declining. This elevated firing rate demonstrated capsaicin’s potent enhancement of mesenteric afferent responsiveness (Figure 2(h)). Subsequently, capsaicin was used to explore whether the action of EA treatment would be affected. Following long‐term EA treatment, the sensitivity of visceral motility to capsaicin was reduced, as evidenced by a decrease in the chemical sensitivity of neural discharge (the spike rate of mesenteric afferent was decreased from 10,000 imp to 2500 imp). Despite continued excitation of afferent nerves by direct stimulation of TRPV1 on mesenteric afferents, the intensity of excitation was lower, suggesting that EA decreased the chemosensitivity of mesenteric afferents.

To determine whether EA‐influenced changes in visceral hypersensitivity are mediated by TRPV1, we administered a TRPV1 agonist or antagonist locally in the intestine and recorded EMG data (Figure 2(i)). Both the FD group and capsaicin induced visceral hypersensitivity in noxious pressures (Figure 2(j)). The visceromotor response was weakened triggered by TRPV1 antagonist SB366791 injection. The selectivity of the TRPV1 antagonist SB366791 was confirmed through EMG signal analysis, indicating a loss of TRPV1 sensitivity within the local intestinal region. EA reduced the visceromotor response induced by noxious pressures (40, 60, 80 mmHg). The application of EA could decrease the visceral hypersensitivity, while the effect of EA was significantly diminished following capsaicin administration. The heightened visceromotor response triggered by capsaicin injection aligned with expectations. These data, taken together with in vitro mesenteric discharge data, indicated that expression of TRPV1 located on sensory afferents was decreased by EA treatment.

### 3.4. EA at ST36 Regulated Enteric Neuron by Decreasing Eosinophils Degranulation

The baseline presence of resident eosinophils in the duodenal immune composition was minimal (Figure 3(a)). Eosinophil infiltration to the mucosa and submucosal layers was not altered in the Con group (the density of eosinophils was nearly 15%) (Figures 3(a) and 3(b)). Duodenal eosinophils infiltration has been notably observed in the FD group (eosinophils infiltration rose from 15% to 40%). The density of eosinophils in the duodenal area was influenced by EA treatment, with a reduction (eosinophil infiltration was reduced from 40% to 10%) in immune cell infiltration. As expected, the SEA group did not have similar anti‐inflammatory effects that the density of eosinophils maintained the high level.

FIGURE 3EA at ST36 decreased eosinophils degranulation. (a) Eosinophils stained by chromotrope 2R in the duodenum. Representative pictures of the duodenum. Eosinophils are indicated by arrows. (b) The number of lamina propria eosinophils. ^∗^ *p* < 0.05; ^∗∗^ *p* < 0.01; ^∗∗∗^ *p* < 0.001. *n* = 3‐6 per group. (c) Representative images showing immunolabeling for EMBP in rat duodenum. (d) The number of EMBP cells in duodenum. ^∗^ *p* < 0.05; ^∗∗^ *p* < 0.01; ^∗∗∗^ *p* < 0.001. *n* = 3–6 per group. (e and f) Quantification of local EMBP production within the rat duodenum by western blotting. *n* = 3 per group.

**Figure:**
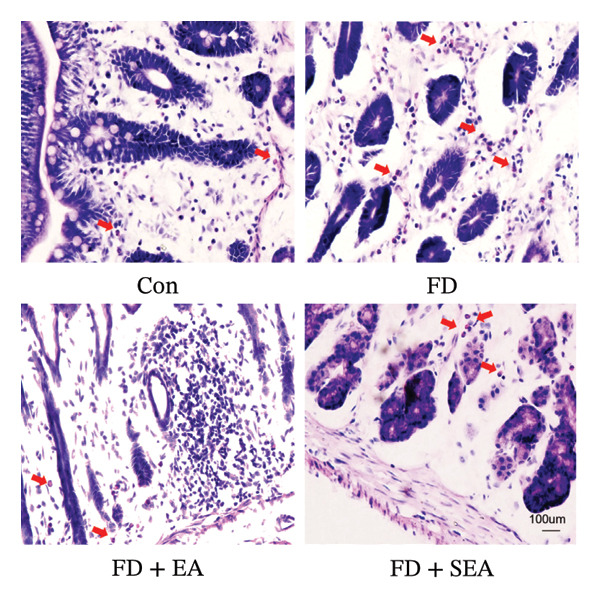
(a)

**Figure:**
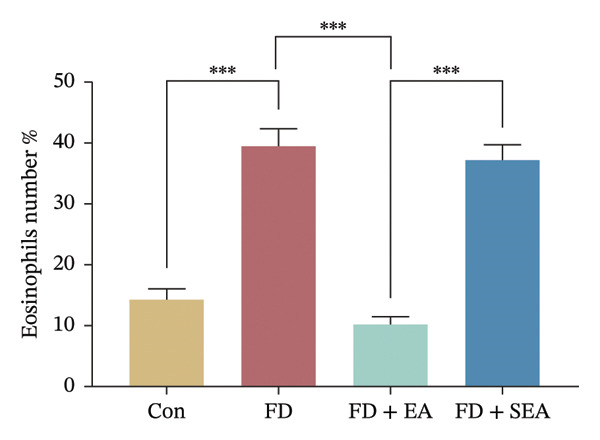
(b)

**Figure:**
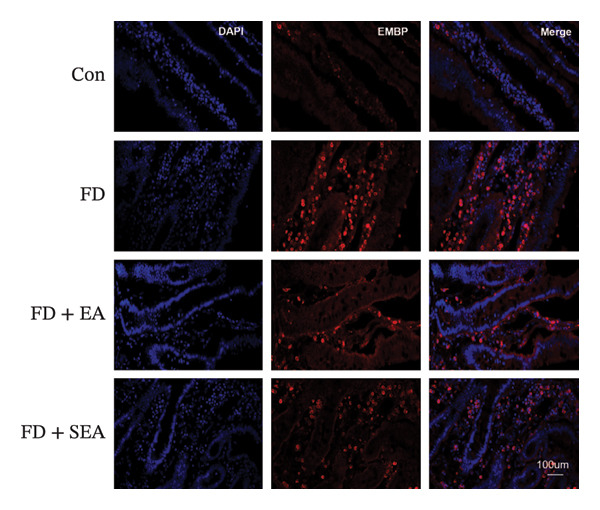
(c)

**Figure:**
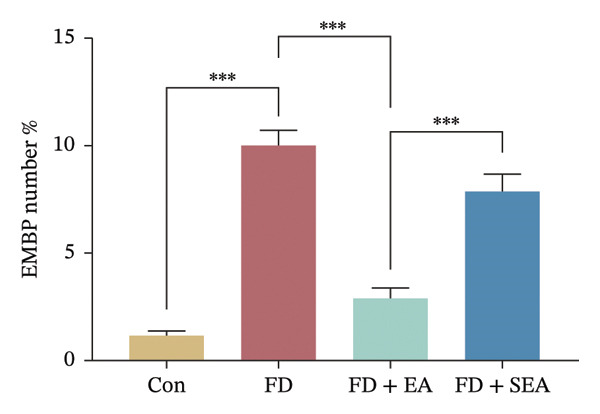
(d)

**Figure:**
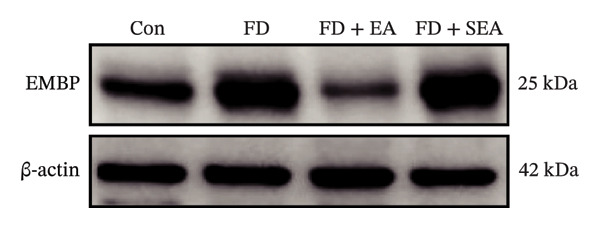
(e)

**Figure:**
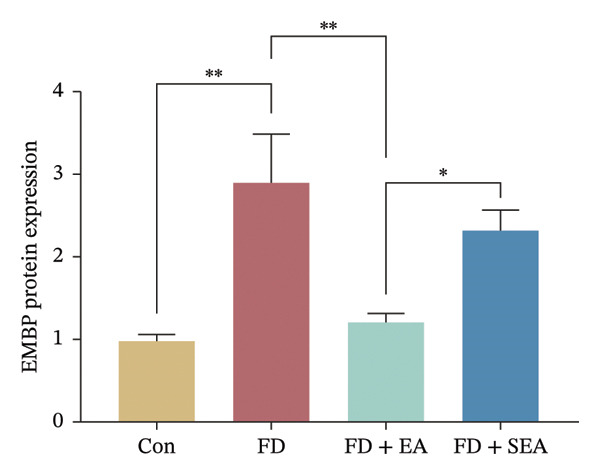
(f)

The identification of eosinophils and subsequent exploration of their functional roles are closely tied to the diverse groups of proteins stored within the abundant secondary granules located in the cytoplasm of these immune cells. The distribution of EMBP was predominantly observed in the duodenal submucosa and lamina propria. Eosinophils were found to be elevated in the duodenal tissue in the FD group, accompanied by changes in degranulation indicative of activation (Figures 3(c) and 3(d)). Duodenal eosinophils degranulation has been notably observed in the FD group (the number of degranulation rose from 1.5% to 10%). EMBP immunoreactivity in the submucosal layers of the EA group (2.5%) was significantly lower in comparison to the FD group. SEA treatment did not contribute to a notable reduction in EMBP density compared to the EA group. In agreement with these changes in immunoreactivity image, EMBP protein levels in the duodenum from the FD group were profoundly increased (Figures 3(e) and 3(f)). Importantly, the EA group exhibited lower EMBP expression, while protein levels still maintained high expression after SEA treatment. In brief, these findings demonstrated that EA, rather than SEA, exerted marked anti‐inflammatory effects on duodenal microinflammation.

EMBP labeling in the submucosal layers and lamina propria was localized to the close proximity to enteric nerve terminals, as indicated by co‐staining with the pan‐neuron marker PGP9.5‐positive fibers (Figure 4(a)). This observation suggested a potential immune–nerve interaction in the duodenum. To confirm whether the enteric neurons in the duodenum are affected by EMBP, we conducted primary culture of enteric neuron derived from the rat duodenum (Figure 4(b)). Stimulation of enteric neuron cultures with purified EMBP protein resulted in an obvious increase in the length of neuron fibers (Figures 4(c) and 4(d)). The length of enteric neuron fibers was increased following stimulation with both low and high doses of EMBP (Figure 4(d)). Furthermore, we observed that the expression of TRPV1 receptors was present on neurons following stimulation with EMBP (Figure 4(e)).

FIGURE 4EA at ST36 regulated enteric neuron by decreasing eosinophils degranulation. (a) Representative images of immunolabeling for enteric neuron (labeled with PGP9.5, red) and eosinophils degranulation mediators (labeled with EMBP, green) in the enteric system of the four group of duodenum. Scale bars, 20 μm, 40X. (b) Schematic of the primary enteric neuron culture experiment by Figdraw. (c) Culture and differentiation of enteric neuron (labeled with tubulin, red). Stimulation by different dose of EMBP. (d) The length of Tubulin neuron. ^∗^ *p* < 0.05; ^∗∗^ *p* < 0.01; *n* = 12 rats. (e) Representative images of immunolabeling for Tubulin (green) and TRPV1 receptors (red) in the enteric system of the four groups of duodenal. Scale bars, 20 μm, 40X. (f) The co‐labeled Tubulin and TRPV1.

**Figure:**
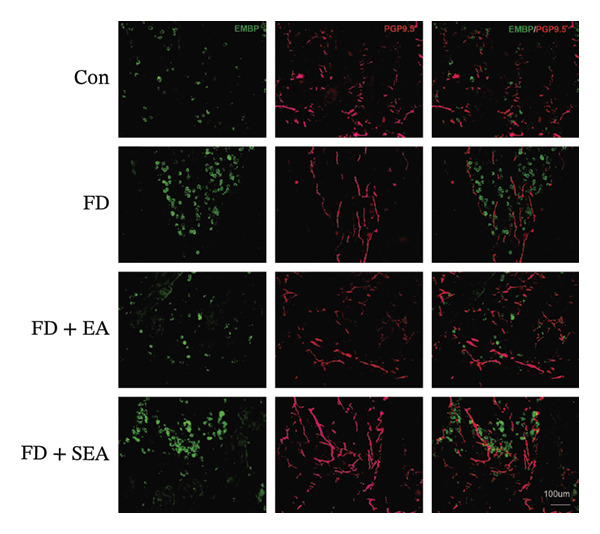
(a)

**Figure:**
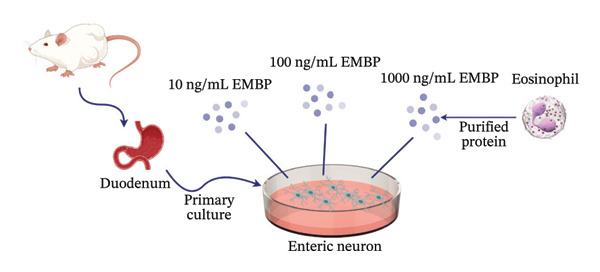
(b)

**Figure:**
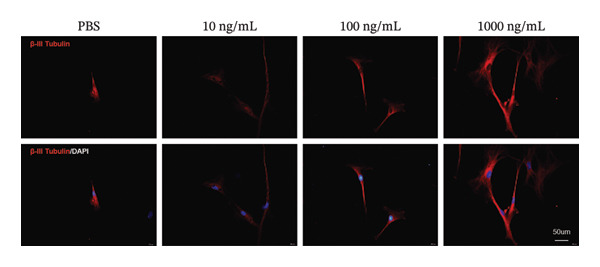
(c)

**Figure:**
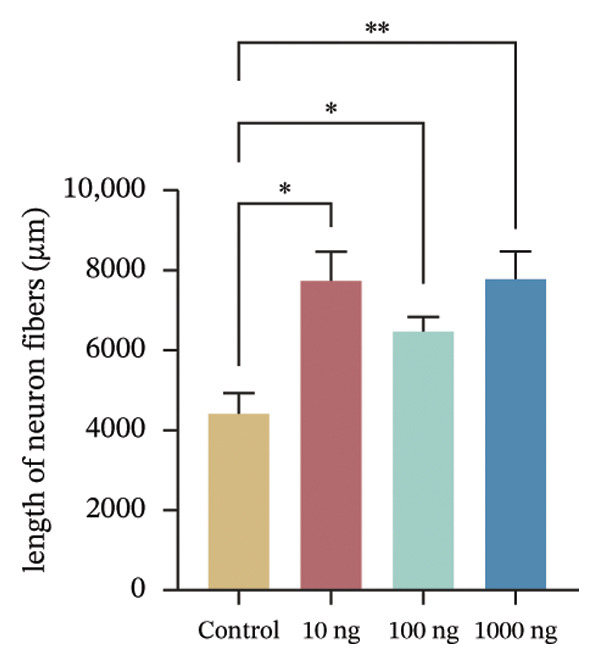
(d)

**Figure:**
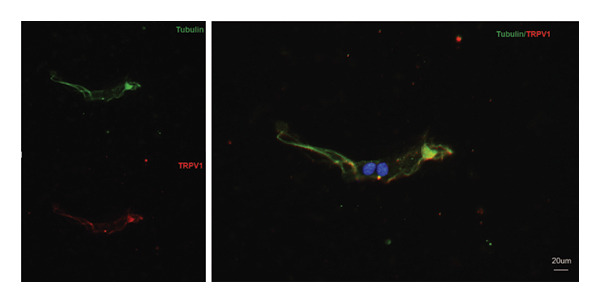
(e)

**Figure:**
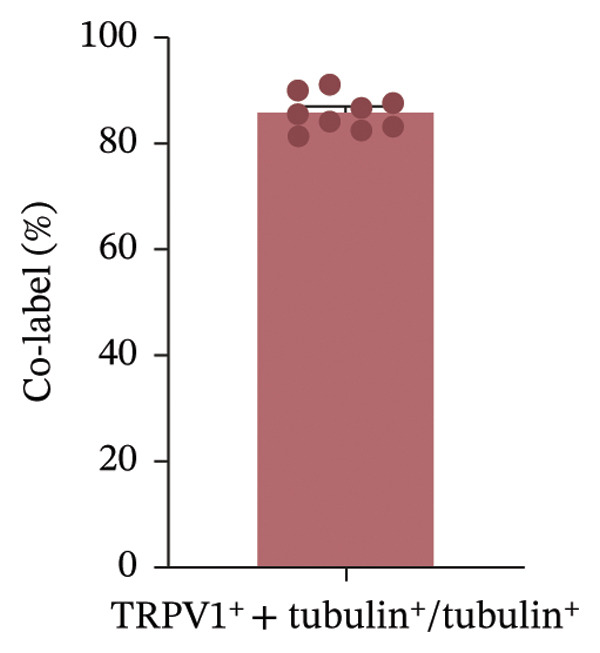
(f)

## 4. Discussion

**Figure 5:**
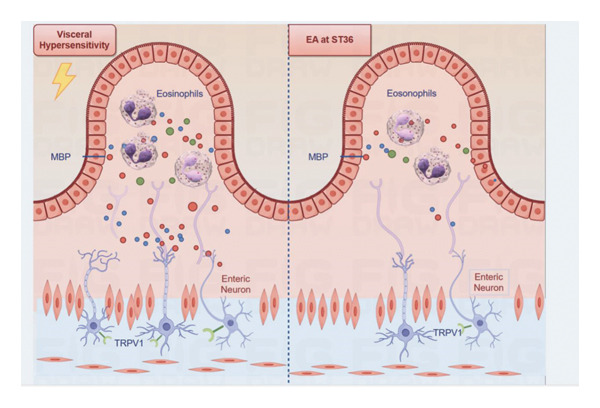
Abstract by Figdraw.

The findings presented in this study demonstrate that electroacupuncture is a potentially effective treatment for visceral hypersensitivity. Our investigation revealed that EA significantly alleviates the increased sensitivity of visceral nerve pathways and lowers the expression levels of TRPV1 receptors. Furthermore, a reduction in eosinophil activation in response to EA suggests that EA was a therapeutic strategy for inhibiting the development of visceral hypersensitivity by regulating communication between eosinophils and enteric neurons (see Figure 5).

The manifestation of gastrointestinal symptoms primarily results from the activation of specialized sensory receptors including chemosensitive and mechanosensitive nociceptors within the digestive system [23]. The TRPV1 receptors, which are extensively distributed on sensory nerves and visceral organ, are involved in inducing gastrointestinal symptom [24, 25]. Our results indicate that intestinal neural sensitization, characterized by increased expression of TRPV1 receptors, is a key contributor to visceral hypersensitivity. The increased expression of TRPV1 receptors likely leads to heightened sensitivity of enteric neurons to various stimuli, resulting in the exaggerated perception of visceral sensations as painful or uncomfortable. This downregulation of TRPV1 receptors may be a critical mechanism by which EA alleviates visceral hypersensitivity. EA diminishes the ability of these neurons to be activated by noxious stimuli, thereby lowering the overall level of visceral sensitivity. This finding aligns with previous studies demonstrating the involvement of TRPV1 receptors in various pain conditions and the therapeutic potential of targeting these receptors for pain management. EA at ST36 relieves visceral hypersensitivity via the mast cell–triggered TRPV1 peripheral afferent pathway in post‐inflammation rats [26]. Recently, Liu and colleagues have demonstrated that high‐intensity EA applied to deep tissue at the lower limb acupoint ST36 enhances gastric motility in both healthy subjects and patients with FD presenting with motor dysfunction symptoms [27]. Specifically, high‐intensity EA targeting deep but not superficial lower limb tissues activates TRPV1‐expressing somatosensory afferent nerves. These afferents subsequently stimulate vagal efferent neurons, which project onto choline acetyltransferase‐positive cholinergic enteric neurons, ultimately modulating gastric function. Studies utilizing ultrasound imaging to guide needle insertion depth have indicated a strong correlation between acupuncture‐evoked sensations and tissue depth [28]. Consistent with this, our study revealed that the EA exhibited reduced duodenal TRPV1 expression. This effect may be attributed to deep needling at ST36 in the EA group, which activates the somatosensory system innervating deep tissues and thereby ameliorates visceral hypersensitivity.

Eosinophils, a type of immune cell typically associated with allergic responses, have been increasingly recognized for their involvement in gastrointestinal disorders [8, 9]. Eosinophil degranulation, the release of toxic granule proteins from eosinophils, can lead to tissue damage, inflammation, and nerve sensitization [29]. Research evidence has demonstrated that enteric nervous system abnormalities, characterized by eosinophil aggregation, are consistently observed in mucosal specimens obtained from individuals diagnosed with functional gastrointestinal disorders [8, 9]. Specifically, investigations focusing on FD have revealed significant neuronal dysfunction within the submucosal plexus of duodenal biopsy samples. These pathological changes are manifested through upregulated expression of glial cell markers, structural and functional alterations in ganglionic organization, along with enhanced cellular infiltration involving both eosinophils and mast cells in the submucosal neural network [8]. This evidence supports the idea that the duodenal submucosal nerve endings are activated by eosinophils, potentially leading to persistent alterations in the structure, function, and connectivity of the neuronal network. Our data demonstrate that eosinophils were activated and degranulating in the intestinal tissue of FD rats, releasing substances that directly stimulated enteric neurons, thus contributing to their sensitization. We found that EA treatment significantly reduced eosinophil activation and degranulation in the gut. By inhibiting the release of these sensitizing substances, EA may interrupt the feedback loop of eosinophil–nerve interaction, thereby reducing visceral sensitivity. Some studies have demonstrated that acupuncture exerts significant anti‐inflammatory effects through regulating specific classical inflammatory cells, cytokines, and cellular signaling pathways [30, 31]. These findings indicate that stimulation of ST36 acupoint can modulate the recruitment and degranulation of eosinophils in the duodenum. Several studies have demonstrated that EA at ST36 is frequently utilized for the management of immunological disorders, as it regulates intestinal T lymphocytes and attenuates sepsis‐induced systemic inflammatory responses [30, 32, 33]. Experimental studies on irritable bowel syndrome (IBS) revealed that EA applied at the ST36 acupoint significantly attenuates mast cell–derived interleukin‐1β (IL‐1β) and IL‐8 production in colonic tissues, while concurrently ameliorating visceral hypersensitivity [34].

Eosinophils play an essential role in regulating enteric neurons, and this bidirectional interaction is crucial for gastrointestinal tract function [35]. Eosinophils possess the capacity to modulate the ENS by regulating paracrine signals among diverse inflammatory cells within the intestinal mucosa, lamina propria, and smooth muscle [36]. We propose that the degranulation products released by eosinophils act directly on enteric neurons, causing them to become sensitized and more responsive to stimuli. This interaction can lead to the production of visceral pain when stimulated by inflammatory factors, causing depolarization of sensory neurons located in the intermuscular or submucosal plexus of the colon during visceral hypersensitivity [37]. Furthermore, the presence of inflammatory mediators released by eosinophils may enhance the expression of TRPV1 receptors on enteric neurons, further amplifying the cycle of sensitization and pain perception [38]. By reducing eosinophil degranulation and the subsequent release of sensitizing substances, EA may indirectly affect TRPV1 receptor expression and neuronal excitability. EA may be involved in reducing the visceral hypersensitivity through these mechanisms. Studies have speculated that acupoint stimulation activates TRPV1 channels in acupoint tissue, which initiates acupoint signal transduction. Subsequently, this process inhibits the activation of TRPV1 channel in the colon, reduces intestinal hypersensitivity, modulates colon motility, and improves abdominal symptoms [39]. This synergistic effect of EA on both eosinophils and enteric neurons underscores the complex interplay between immune and nervous system components in the pathophysiology of FD. Nonetheless, the current research has not yet fully elucidated the complete mechanism of EA action. Other immune cells and molecular pathways, beyond the eosinophils involved in enteric nervous system, are expected to contribute to visceral hypersensitivity in FGIDs [40].

Our data demonstrated that while SEA elicited neuromodulatory effects on electrophysiological recordings similar to those of EA, it failed to induce significant improvement in key pathological markers such as EMBP levels in the FD model. These results suggested that SEA, likely acting through nonspecific cutaneous sensory input, could evoke generalized neural responses but is insufficient to engage the downstream immunomodulatory and molecular changes required for substantial therapeutic reversal [41]. Mechanistically, this difference might be attributed to the stimulation depth and the distinct neural substrates activated. It has been shown that deep fascial tissues were innervated by neurons that differ from those supplying the superficial epidermal layers. Low‐intensity stimulation of superficial tissue might not sufficiently recruit the neural circuits necessary to inhibit inflammatory responses effectively [17, 18]. Such divergence in treatment outcomes could stem from differences in mechanoreceptors, nociceptors, and the distribution of TRP channels across tissue layers. Furthermore, afferent signals originating from different tissue depths at the same acupoint may project to distinct spinal segments, thereby activating separate central pathways [42]. Consequently, acupuncture effects can vary significantly depending on the depth of needle insertion [41]. Collectively, this evidence indicates that SEA is not a biologically inert intervention but rather a partially active control that engages nonspecific neural pathways. The acupoint specificity and optimized stimulation parameters of EA are essential to activate the integrated neuro‐immune circuit that leads to sustained therapeutic efficacy. A potential limitation of our study lies in the elucidation of the precise causal relationship between eosinophil suppression and TRPV1 modulation following EA at ST36. Our data showed that EA reduced both eosinophil infiltration and TRPV1‐mediated neuronal sensitization. In vitro evidence further indicated that the eosinophil‐derived mediator EMBP can sensitize neurons. However, our current experimental design could not definitively distinguish whether the decrease in TRPV1 activity results directly from reduced eosinophil signaling, or whether it arises from other, independent mechanisms concurrently activated by EA. It also remained possible that EA acted on both the immune and neuronal pathways simultaneously [43]. Alternatively, the modulation of TRPV1 might have preceded or occurred independently of changes in eosinophil activity. Future studies employing cell‐specific depletion or inhibition strategies in vivo, or time‐course experiments tracking earlier molecular events, would be valuable to establish a clearer directional causality. While these experiments provided some evidence that eosinophil‐derived mediators could sensitize intestinal neurons, certain aspects warrant consideration for interpretation. The concentration and exposure time of EMBP used were selected based on preliminary dose–response experiments and aimed to simulate a pathophysiological, rather than physiological status, state akin to that suspected during FD. However, the precise in vivo concentration of EMBP and other granule proteins within the duodenal mucosal microenvironment in our model remained unquantified [44]. The simplified culture system, while invaluable for isolating direct neuron–eosinophil mediator interactions, inherently lacked the integrated tissue context present in vivo, including the epithelial barrier, resident immune cells, and concurrent neural network activity. Therefore, future studies measuring local mediator levels and employing more complex co‐culture systems or targeted in vivo neutralization would help to solidify the relevance of this mechanism. Although this study focused on enteric neuron–eosinophil interactions, we recognize that enteric glia and other immune populations may substantially contribute to inflammatory cascades, neuroinflammatory processes, and visceral sensitivity modulation [45]. Furthermore, it remains unclear whether observed changes in local intestinal indicators result from the early‐stage blockade of incoming pain signals or the influence of analgesic signals originating from higher centers mediated by EA. Exploring the specific interactions of EA across different pathways can open new avenues for future studies. Future studies are needed to determine which of these signaling molecules and neuron types are involved in EA treatment of immune‐nerve‐mediated visceral mechanical or sensory hypersensitivity.

## 5. Conclusion

In summary, our study elucidates the beneficial effects of electroacupuncture in reducing visceral hypersensitivity in FD. By targeting key mechanisms such as neural sensitization through TRPV1 downregulation and eosinophil activation reduction, electroacupuncture demonstrates promise as a therapeutic strategy for managing FD.

## Author Contributions

Cun‐Zhi Liu conceptualized the study. Yue‐Jie Li performed all experiments. Lu‐Ping Liu and Na‐Na Yang summarized the data analysis and figures. Jing‐Wen Yang revised the manuscript.

## Funding

This work was supported by the National Key Research And Development Program of China (No. 2022YFC3500602) and sponsored by Beijing Nova Program (No. 20250484776).

## Disclosure

All authors have approved the submitted version.

## Ethics Statement

All animal experiments were approved by Institutional Animal Care and Use Committee (BUCM‐4‐2022021803‐1021).

## Consent

The authors have nothing to report.

## Conflicts of Interest

The authors declare no conflicts of interest.

## Data Availability

The datasets used during the present study are available from the corresponding author upon reasonable request.
